# Systemic inflammatory markers of persistent cerebral edema after aneurysmal subarachnoid hemorrhage

**DOI:** 10.1186/s12974-022-02564-1

**Published:** 2022-08-04

**Authors:** Sung-Ho Ahn, Angela Burkett, Atzhiry Paz, Jude P. Savarraj, Sarah Hinds, Georgene Hergenroeder, Aaron M. Gusdon, Xuefeng Ren, Jeong-Ho Hong, Huimahn A. Choi

**Affiliations:** 1 Department of Neurology, Pusan National University School of Medicine, Research Institute for Convergence of Biomedical Science and Technology, Pusan National University Yangsan Hospital, Yangsan-si, South Korea; 2 Division of Neurocritical Care, Department of Neurosurgery, University of Texas Health Science Center at Houston, 6431 Fannin, MSB 7.154, Houston, TX 77030 USA; 3 Department of Neurology, Keimyung University School of Medicine, Dongsan Medical Center, Daegu, South Korea

**Keywords:** Subarachnoid hemorrhage, Cerebral edema, Cytokine

## Abstract

**Background:**

Cerebral edema (CE) at admission is a surrogate marker of ‘early brain injury’ (EBI) after subarachnoid hemorrhage (SAH). Only recently has the focus on the changes in CE after SAH such as delayed resolution or newly developed CE been examined. Among several factors, an early systemic inflammatory response has been shown to be associated with CE. We investigate inflammatory markers in subjects with early CE which does not resolve, i.e., persistent CE after SAH.

**Methods:**

Computed tomography scans of SAH patients were graded at admission and at 7 days after SAH for CE using the 0–4 ‘subarachnoid hemorrhage early brain edema score’ (SEBES). SEBES ≤ 2 and SEBES ≥ 3 were considered good and poor grade, respectively. Serum samples from the same subject cohort were collected at 4 time periods (at < 24 h [T1], at 24 to 48 h [T2]. 3–5 days [T3] and 6–8 days [T4] post-admission) and concentration levels of 17 cytokines (implicated in peripheral inflammatory processes) were measured by multiplex immunoassay. Multivariable logistic regression analyses were step-wisely performed to identify cytokines independently associated with persistent CE adjusting for covariables including age, sex and past medical history (model 1), and additional inclusion of clinical and radiographic severity of SAH and treatment modality (model 2).

**Results:**

Of the 135 patients enrolled in the study, 21 of 135 subjects (15.6%) showed a persistently poor SEBES grade. In multivariate model 1, higher Eotaxin (at T1 and T4), sCD40L (at T4), IL-6 (at T1 and T3) and TNF-α (at T4) were independently associated with persistent CE. In multivariate model 2, Eotaxin (at T4: odds ratio [OR] = 1.019, 95% confidence interval [CI] = 1.002–1.035) and possibly PDGF-AA (at T4), sCD40L (at T4), and TNF-α (at T4) was associated with persistent CE.

**Conclusions:**

We identified serum cytokines at different time points that were independently associated with persistent CE. Specifically, persistent elevations of Eotaxin is associated with persistent CE after SAH.

**Supplementary Information:**

The online version contains supplementary material available at 10.1186/s12974-022-02564-1.

## Background

Subarachnoid hemorrhage (SAH) continues to be associated with high rates of morbidity in survivors [1, 2]. Early brain injury (EBI)—typically referred to as injurious processes occurring within 72 h after aneurysm rupture—have become a key area of SAH research [3, 4]. Clinical and pre-clinical studies have shown that several pathophysiological processes including early vasospasm, inflammation, and cerebral edema (CE) occur during this phase [5–7]. CE is an important surrogate marker of EBI and CE at admission is associated with secondary complications and poor outcomes [8, 9]. We have previously proposed a clinically practical scale called the ‘subarachnoid hemorrhage early brain edema Score (SEBES)’ and demonstrated that SEBES can predict secondary complications and poor outcomes [10]. Studies have validated SEBES in external patient cohorts [11–13] .

Until recently CE after SAH has been studied as a static process which occurs early on after aneurysm rupture. Little emphasis has been given to the changes in CE after SAH. Using the SEBES scale it has been showed that delayed CE resolution after SAH is an important marker of ongoing injury which as an impact on outcomes [14]. The causes of impaired CE resolution have not been explored. SAH induces a peripheral immune response and activated peripheral immune cells are recruited to the brain parenchyma, where they release cytokines that induce upregulation of intrinsic receptors leading to widespread neuroinflammation [15, 16].

We hypothesize that on-going systemic inflammation is a factor that contributes to persistent CE [17, 18]. The primary goal of this study is to identify systemic inflammatory markers associated with impairments in the resolution of CE. We quantified CE longitudinally using the SEBES score and classified patients as those with and without impairments in CE resolution. In the same cohort of patients, we serially measured systemic levels of several inflammatory proteins from acute to subacute phase of SAH and identified independent cytokines contributing, i.e., persistent CE after adjusting clinically relevant variables especially including age considering well-known relationship with degree of CE. In addition, we examined the associations between clinical course and changes in CE across time.

## Methods

### Study patients

We included patients who were diagnosed with acute SAH and admitted to the Neuroscience Intensive Care Unit at the Memorial Herman Hospital-Texas Medical Center, Houston, TX between July 2013 and March 2019 in an ongoing prospective, observational, single center cohort study. Inclusion criteria were: (a) age > 18 years and (b) spontaneous aneurysmal SAH diagnosed by computed tomography (CT) within 24 h of ictus or by xanthochromia in cerebrospinal fluid if the CT was not diagnostic. Exclusion criteria were: (a) non-aneurysmal SAH including due to trauma, arteriovenous malformation and mycotic aneurysms and (b) comorbidities that could affect baseline inflammation including autoimmune diseases, suspicion of infection, history of malignancy, and current pregnancy. Institutional review board approval was obtained (HSC-MS-12-0637) and written informed consent was obtained from the patient or surrogate.

### Clinical and imaging data

We collected clinical data included age, gender, medical and social history, clinical status at admission including neurological status based on the World Federation of Neurosurgical Societies scale (WFNS) grade [19] and loss of consciousness at ictus. Image data were adjudicated for the presence of intraventricular hemorrhage (IVH), hydrocephalus (based on clinical adjudication) and cerebral infarction, and were independently evaluated by a study neurologist for the amount and location of blood as categorized by the modified Fisher score (mFS). Presence and severity of angiographic vasospasm were assessed by visual inspection of cerebral angiography 7 days after SAH. Angiographic vasospasm was defined as narrowing of the arterial vessel lumen, with severe vasospasm as narrowing exceeding 50% of the normal caliber.

### CE based on the SEBES grade

All cerebral CT scans from admission to 6–8 days were graded for SEBES by 2 independent raters blinded to clinical data. SEBES is a semiquantitative score that measures CE on cranial CT on the basis of two CT levels and can be used to predict outcome after SAH. When there was a discrepancy the CT was graded by a third rater. The SEBES was assessed by assigning one point for the absence of visible sulci either because of effacement of sulci or loss of gray–white differentiation at 2 predetermined levels in each hemisphere: (1) at the level of the insular cortex, where the thalamus and basal ganglion are visible, above the basal cistern and (2) at the level of the centrum semiovale above the level of the lateral ventricle. Patients were dichotomized by non-impaired (SEBES ≤ 2 in follow-up CT) and poor (SEBES ≥ 3 at) CE resolution cohorts.

In patients where high-grade SEBES (3 or 4 points) was detected, the SEBES grade was determined on all subsequent CT scans of the brain at 5–7 days after SAH. All available head CT scans performed according to clinical necessity were examined. Based on the baseline and follow-up SEBES grading, we defined persistent CE as poor SEBES grade at both baseline and follow-up periods**.**

### Outcome definitions

Definition of delayed cerebral ischemia (DCI) was adhered to the proposed definition as “the occurrence of focal neurological impairment (such as hemiparesis, aphasia, apraxia, hemianopia, or neglect), or a decrease of at least 2 points on the Glasgow Coma Scale (either on the total score or on one of its individual components [eye, motor on either side, verbal]), which should be last for at least 1 h and not be apparent immediately after aneurysm occlusion, and attributed to other causes by means of clinical assessment, CT or magnetic resonance imaging (MRI) scanning of the brain, and appropriate laboratory studies” [20]. We adjudicated a DCI through consensus of at least two attending neurointensivists in weekly research meetings. When no consensus can be achieved, the principal investigator made the final determination [21]. Long-term functional outcome was assessed by the modified Rankin Scale (mRS) at 3 months after SAH, and a mRS of 4 to 6 was defined as unfavorable outcomes. Subjects were treated according to standard guidelines [22].

### Sample collection and processing protocol

Patients’ serum samples were collected at 4 predetermined time points from admission to subacute stage after SAH: < 24 h (T_1_), between 24 and 48 h (T_2_), 3–5 days (T_3_) and 6–8 days (T_4_). A total of 17 serum cytokine concentrations in pg/mL were measured following manufacturer’s protocol using a MAGPIX magnetic bead based ELISA17-plex assay (EMD Millipore, Billerica, MA): Eotaxin/CCL11; GCSF; GM-CSF; IFN- γ; IL-10; IL-1Ra; IL-6; IL-8; IP-10; MCP-1; MIP1-α; MIP1-β; TNF-α; sCD40L; RANTES; PDGF-AA; PDGF-AB/BB. They were analyzed based on previously published protocols (see Additional file 1) [23].

### Statistical analysis

Study subjects’ characteristics were described using unpaired Student’s *t* test or non-parametric Mann–Whitney *U* test for continuous variables and Pearson chi-square test or non-parametric Fisher’s exact test for categorical variables. In addition, characteristics of subgroups according to the change of CE were described using unpaired ANOVA test or non-parametric Kruskal–Wallis test for continuous variables and Pearson chi-square test or non-parametric Fisher’s exact test for categorical variables. For screening of candidate cytokines possibly associated with persistent CE, the non-parametric Mann–Whitney *U* test (*p* < 0.20) was used to identify candidate cytokines in each time period that differentiated persistent CE based on the revised method of previous research [24]. Using these candidate cytokines, multivariate logistic regression analyses were step-wisely performed to find cytokines independently associated with persistent CE adjusting for main clinical variables including age, sex and past medical history (model 1), and additional inclusion of clinical and radiographic severity of SAH and treatment modality (model 2). The results of the multivariate logistic regression analysis were reported as an odds ratio (OR) at a 95% confidence interval (CI). A *p* value of ≤ 0.05 was considered statistically significant (two-tailed). All statistical analyses were performed using SPSS 17.0 (SPSS Inc., Chicago, Ill).

## Results

### General characteristics

During the study period, 261 patients with acute SAH were admitted to the Neuroscience Intensive Care Unit at the Memorial Herman Hospital-Texas Medical Center, Houston, TX. To maintain homogeneity, among patients who met the inclusion criteria, only 135 subjects who had at least 2 blood draws over time were included in this study.

The mean age of the subjects was 52.2 ± 12.8 years (range 20 to 87 years) and 104 (77.0%) were women. Overall, 90 subjects had their aneurysm clipped, 43 had embolization with coils and 2 subjects were untreated. Based on baseline and follow-up SEBES grading, 21 of 135 subjects (15.6%) showed a persistently poor SEBES grade. Subjects with persistent CE had higher WFNS grade than those without persistent CE (Table 1).

**Table 1:** Clinical variables in patients according to presence of persistent CE

Variable	Classified by baseline and follow-up SEBES grade		
	Persistently poor (*n* = 21)	Others (*n* = 114)	*p *value^†^
Age (years)	48.8 ± 11.2	52.9 ± 13.0	0.18
Gender, male	3 (14.3)	28 (24.6)	0.40
Race			0.84
White	15 (71.4)	74 (64.9)	
Black	4 (19.0)	27 (23.7)	
Asian or others	2 (9.5)	13 (11.4)	
Risk factors and comorbidities			
Hypertension	12 (57.1)	78 (68.4)	0.31
Dyslipidemia	1 (4.8)	15 (13.2)	0.47
Diabetes mellitus	2 (9.5)	17 (14.9)	0.74
Current smoking	4 (19.0)	46 (40.4)	0.09
Prior coronary artery disease	1 (4.8)	11 (9.6)	0.69
Prior stroke	0 (0.0)	11 (9.6)	0.21
Clinical and radiographic severity of SAH			
WFNS grade	4 [3–5]	2 [1–4]	< 0.01
mFS grade	3 [3–3]	3 [3–3]	0.91
IVH	14 (66.7)	75 (65.8)	0.94
Hydrocephalus	17 (81.0)	73 (64.0)	0.13
Therapeutic intervention			
Coiling	8 (38.1)	35 (30.7)	0.50

Variables are presented as mean ± SD, median [interquartile range], or number (%)

*CE* cerebral edema, *IVH* intraventricular hemorrhage, *mFS* modified Fisher, *SAH* subarachnoid hemorrhage, *SEBES* subarachnoid hemorrhage early brain edema score, *WFNS* World Federation of Neurological Surgeons

^†^*p* value are calculated by Pearson chi-square test or Fisher’s exact test, or Student’s *t* test or Mann–Whitney *U* test as appropriate

### Cytokines associated with persistent CE

At T_1_, T_2_, T_3_ and T_4_, the levels of 5, 4, 3 and 7 cytokines, respectively, were different across the persistent CE, and initially selected as candidate cytokines contributing to persistent CE (Table 2).

**Table 2:** Cytokines associated with CE in each time point

	Persistent CE			
	CE+ (vs CE−)			
Cytokines	T_1_ 19 (vs 108)	T_2_ 18 (vs 98)	T_3_ 20 (vs 102)	T_4_ 19 (vs 96)
Eotaxin	H	–	H	H
GCSF	H	–	H	–
GM–CSF	–	–	–	–
IFN-γ	–	–	–	–
IL-10	H*	–	–	H*
PDGF-AA	–	L*	–	–
PDGF–ABBB	–	-	–	–
sCD40L	–	L	–	–
IL-1Ra	–	–	–	H*
IL-6	H*	–	–	H*
IL-8	H*	H	H*	H*
IP-10	–	–	–	–
MCP-1	–	H	–	H*
MIP-1a	–	–	H*	–
MIP-1β	–	–	–	–
RANTES	–	–	H	–
TNF-α	–	–	–	H
Total^a^	5	4	3	7

*CE* cerebral edema, *L* low level, *H* high level

*Statistically significant difference in cytokine level with a *p* value < 0.05 by Mann–Whitney *U* test for non-parametric test among candidate cytokine which showed either high (H) or low (L) level of cytokine according to the presence of persistent CE

^a^Number of cytokine which showed either high (H) or low (L) level of cytokine in subjects with persistent CE (versus those without persistent CE) with a *p* value < 0.20 by Mann–Whitney *U* test for non-parametric test in each time point were listed

In Table 3, multivariate logistic regression analyses were step-wisely performed to identify independent cytokine associated with persistent CE. Age, sex, and risk factors and comorbidities were included in model 1, and clinical and radiographic severity of SAH including WFNS and mFS grade, presence of IVH and hydrocephalus, and therapeutic intervention was additionally included into the baseline model 1 (model 2). In multivariate model 1, higher Eotaxin at T_1_ (odds ratio [OR] = 1.016, 95% confidence interval [CI] = 1.002–1.030) and at T_4_ (OR = 1.012, 95% CI = 1.001–1.023), sCD40L at T_4_ (OR = 1.003, 95% CI = 1.001–1.006), IL-6 at T_1_ (OR = 1.015, 95% CI = 1.004–1.026) and T_3_ (OR = 1.018, 95% CI = 1.002–1.034) and TNF-α at T_4_ (OR = 1.117, 95% CI = 1.027–1.214) was independently associated with persistent CE. In addition, GCSF at T_3_, IL-10 at T_1,_ PDGF-AA at T_4,_ IL-1Ra at T_3_, MCP-1 at T_4_, and RANTES at T_3_ tended to be associated with persistent CE. In multivariate model 2, Eotaxin at T_4_ (OR = 1.019, 95% CI = 1.002–1.035) was still associated with persistent CE, and PDGF-AA at T_4_, sCD40L at T_4_, and TNF-α at T_4_ tended to be associated with persistent CE even after adjusting all clinically relevant variables.

**Table 3:** Adjusted odds ratio of cytokines for prediction of persistent CE

Variables	Univariable			Multivariable^a^			Multivariable^b^		
	OR	95% CI	*p* value	OR	95% CI	*p* value	OR	95% CI	*p* value
Eotaxin									
Eotaxin (T_1_), per 1 pg/mL increase	1.011	0.999–1.023	0.084	1.016	1.002–1.030	0.029	1.011	0.996–1.026	0.137
Eotaxin (T_2_), per 1 pg/mL increase	1.002	0.987–1.016	0.815	N/A					
Eotaxin (T_3_), per 1 pg/mL increase	1.009	0.996–1.022	0.169	1.013	0.998–1.023	0.080	1.012	0.995–1.029	0.169
Eotaxin (T_4_), per 1 pg/mL increase	1.009	1.00–1.018	0.063	1.012	1.001–1.023	0.030	1.019	1.002–1.035	0.024
GCSF									
GCSF (T_1_), per 1 pg/mL increase	1.003	0.999–1.008	0.172	1.004	0.999–1.009	0.157	1.003	0.997–1.009	0.280
GCSF (T_2_), per 1 pg/mL increase	0.995	0.985–1.006	0.386	N/A					
GCSF (T_3_), per 1 pg/mL increase	1.004	0.988–1.010	0.154	1.015	1.000–1.030	0.054	1.013	0.994–1.031	0.175
GCSF (T_4_), per 1 pg/mL increase	1.007	1.000–1.014	0.064	1.009	0.996–1.022	0.186	1.007	0.922–1.022	0.366
IL-10									
IL-10 (T_1_), per 1 pg/mL increase	1.003	0.999–1.007	0.168	1.005	0.999–1.011	0.076	1.003	0.996–1.011	0.350
IL-10 (T_2_), per 1 pg/mL increase	0.994	0.974–1.014	0.525	N/A					
IL-10 (T_3_), per 1 pg/mL increase	1.006	0.968–1.046	0.757	N/A					
IL-10 (T_4_), per 1 pg/mL increase	1.004	0.993–1.014	0.499	N/A					
PDGF-AA									
PDGF-AA (T_1_), per 10 pg/mL increase	1.001	1.000–1.001	0.216	N/A					
PDGF-AA (T_2_), per 10 pg/mL increase	0.998	0.996–1.001	0.214	N/A					
PDGF-AA (T_3_), per 10 pg/mL increase	1.000	0.999–1.001	0.849	N/A					
PDGF-AA (T_4_), per 10 pg/mL increase	1.000	1.000–1.001	0.143	1.005	1.000–1.011	0.072	1.006	0.999–1.014	0.075
sCD40L									
sCD40L (T_1_), per 10 pg/mL increase	0.998	0.992–1.003	0.419	N/A					
sCD40L (T_2_), per 10 pg/mL increase	0.993	0.983–1.003	0.173	N/A					
sCD40L (T_3_), per 10 pg/mL increase	0.998	0.995–1.001	0.281	N/A					
sCD40L (T_4_), per 10 pg/mL increase	1.000	1.000–1.000	0.060	1.003	1.001–1.006	0.016	1.003	1.000–1.006	0.053
IL1Ra									
IL1Ra (T_1_), per 1 pg/mL increase	1.001	1.000–1.003	0.102	1.002	1.000–1.004	0.103	1.000	0.998–1.003	0.814
IL1Ra (T_2_), per 1 pg/mL increase	1.001	0.997–1.005	0.531	N/A					
IL1Ra (T_3_), per 1 pg/mL increase	1.002	0.999–1.004	0.144	1.002	1.000–1.005	0.071	1.001	0.998–1.004	0.497
IL1Ra (T_4_), per 1 pg/mL increase	1.000	0.996–1.003	0.917	N/A					
IL-6									
IL-6 (T_1_), per 1 pg/mL increase	1.014	1.005–1.023	0.003	1.015	1.004–1.026	0.006	1.008	0.996–1.021	0.200
IL-6 (T_2_), per 1 pg/mL increase	1.002	0.990–1.015	0.696	N/A					
IL-6 (T_3_), per 1 pg/mL increase	1.014	1.002–1.026	0.024	1.018	1.002–1.034	0.025	1.009	0.991–1.027	0.331
IL-6 (T_4_), per 1 pg/mL increase	1.002	0.996–1.008	0.535	N/A					
IL-8									
IL-8 (T_1_), per 1 pg/mL increase	1.018	1.000–1.037	0.047	1.015	0.995–0.948	0.135	1.013	0.990–1.036	0.276
IL-8 (T_2_), per 1 pg/mL increase	1.018	0.992–1.045	0.177	1.017	0.988–1.047	0.255	1.007	0.972–1.043	0.695
IL-8 (T_3_), per 1 pg/mL increase	1.002	0.999–1.005	0.221	N/A					
IL-8 (T_4_), per 1 pg/mL increase	1.010	0.987–1.034	0.398	N/A					
MCP-1									
MCP-1 (T_1_), per 1 pg/mL increase	1.001	0.999–1.003	0.220	N/A					
MCP-1 (T_2_), per 1 pg/mL increase	1.001	0.997–1.005	0.723	N/A					
MCP-1 (T_3_), per 1 pg/mL increase	1.001	1.000–1.003	0.142	1.001	0.999–1.003	0.181	1.000	0.998–1.002	0.741
MCP-1 (T_4_), per 1 pg/mL increase	1.002	1.000–1.004	0.034	1.002	1.000–1.005	0.077	1.002	0.999–1.004	0.171
MIP1a									
MIP1a (T_1_), per 1 pg/mL increase	0.996	0.939–1.056	0.893	N/A					
MIP1a (T_2_), per 1 pg/mL increase	1.000	0.940–1.063	0.992	N/A					
MIP1a (T_3_), per 1 pg/mL increase	1.013	0.983–1.044	0.393	N/A					
MIP1a (T_4_), per 1 pg/mL increase	0.967	0.874–1.071	0.522	N/A					
RANTES									
RANTES (T_1_), per 1 pg/mL increase	1.000	1.000–1.001	0.733	N/A					
RANTES (T_2_), per 1 pg/mL increase	1.000	0.999–1.001	0.591	N/A					
RANTES (T_3_), per 1 pg/mL increase	1.000	1.000–1.001	0.240	N/A					
RANTES (T_4_), per 1 pg/mL increase	1.000	0.999–1.001	0.983	N/A					
TNF-α									
TNF-α (T_1_), per 1 pg/mL increase	1.014	0.951–1.082	0.672	N/A					
TNF-α (T_2_), per 1 pg/mL increase	0.942	0.841–1.055	0.304	N/A					
TNF-α (T_3_), per 1 pg/mL increase	1.010	0.952–1.072	0.740	N/A					
TNF-α (T_4_), per 1 pg/mL increase	1.081	1.081–1.155	0.022	1.117	1.027–1.214	0.010	1.096	0.999–1.202	0.053

*CE* cerebral edema, *CI* confidence interval, *OR* odds ratio, *SAH* subarachnoid hemorrhage

^a^Adjustments were made for age, gender, race, risk factors and comorbidities in Table 1

^b^Adjustments were made for age, gender, race, risk factors, comorbidities, clinical and radiographic severity of SAH and therapeutic intervention in Table 1

**Fig. 1:**
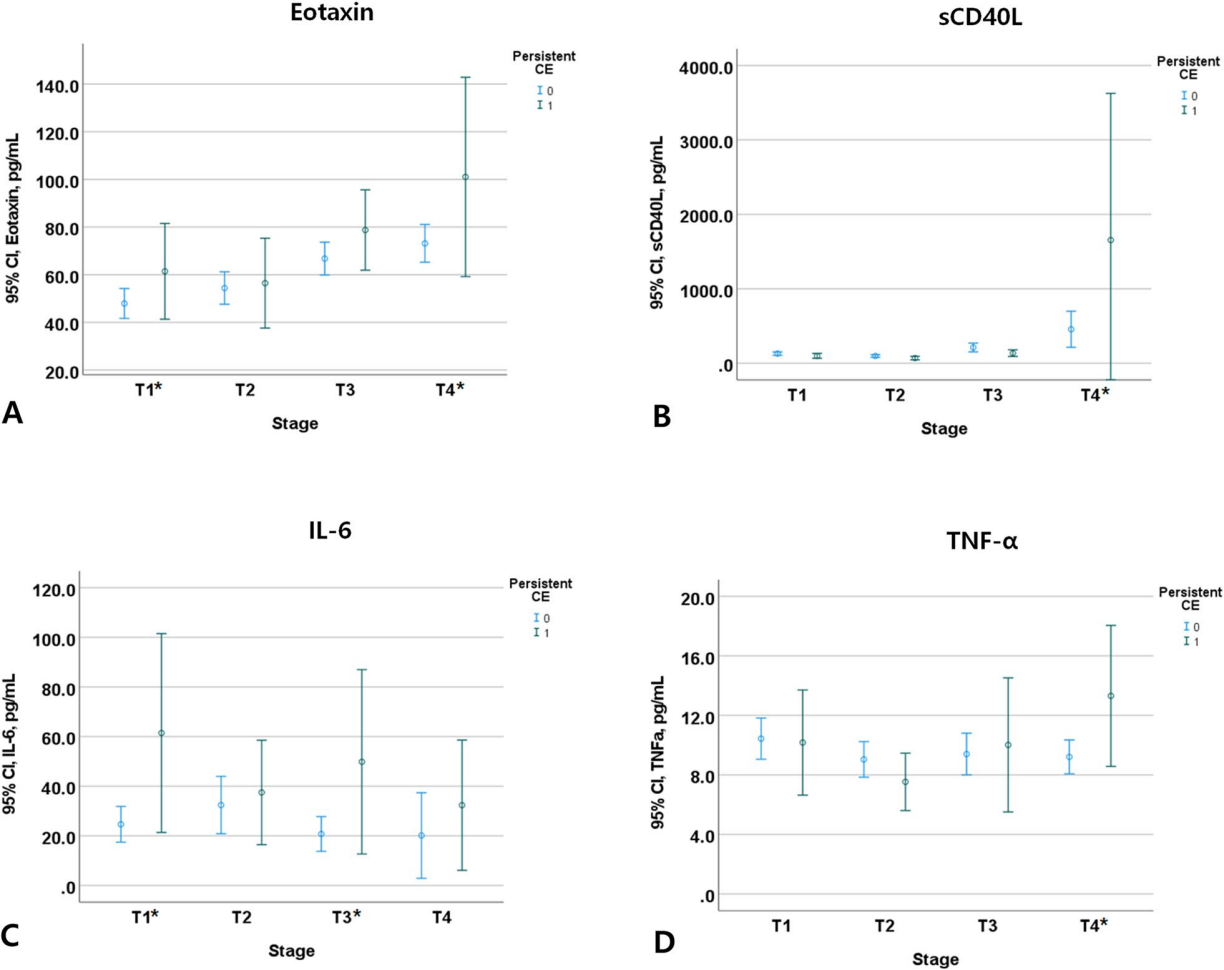
Serum levels of main cytokines across persistent CE. *CE* cerebral edema. *P value < 0.05 was evaluated by the multivariable logistic regression after adjusting age, sex, risk factors and comorbidities

We plotted the trends of values of 4 cytokines that were independently associated with persistent CE in multivariate model 1. Serum levels of Eotaxin and IL-6 were consistently higher and levels of sCD40L and TNF-α were higher especially at T4 stage in persistent CE group compared to other groups (Fig. 1).

### Clinical course according to change of CE

Among enrolled patients, 89 subjects (65.9%) and 43 subjects (31.9%) developed vasospasm and DCI, respectively, all within 7 days after SAH and 31 (23.0%) subjects were reported to have poor functional outcome (mRS ranging from 4 to 6 score at 3 months).

Considering the longitudinal change of CE based on the baseline and follow-up SEBES grading, subjects were classified into four subgroups. Among 41 patients who showed a poor SEBES grade at admission, 21 subjects had a persistently poor SEBES, otherwise 20 subjects showed an improvement in SEBES grade. Among 94 subjects who showed a good SEBES grade at admission, 17 of 94 subjects had a worsened SEBES grade compared to the baseline SEBES grade. Age, WFNS and modified Fisher grade was significantly different among four subgroups (Table 4).

**Table 4:** Clinical variables in patients according to change of CE

Variable	Classified by baseline and follow-up SEBES grade				
	High-grade SEBES, baseline (*N* = 41)		Low-grade SEBES, baseline (*N* = 94)		*p *value^†^
	G4 (persistently poor, *n* = 21)	G3 (improved, *n* = 20)	G2 (worsened, *n* = 17)	G1 (persistently good, *n* = 77)	
Age (years)	48.8 ± 11.2	47.3 ± 10.2	46.8 ± 14.9	55.7 ± 13.5	< 0.01
Gender, male	3 (14.3)	4 (20.0)	2 (11.8)	22 (28.6)	0.29
Race					1.00
White	15 (71.4)	13 (65.0)	12 (64.7)	50 (64.9)	
Black	4 (19.0)	5 (25.0)	4 (23.5)	18 (23.4)	
Asian or others	2 (9.5)	2 (10.0)	2 (11.8)	9 (11.7)	
Risk factors and comorbidities					
Hypertension	12 (57.1)	16 (80.0)	10 (58.8)	52 (67.5)	0.40
Dyslipidemia	1 (4.8)	4 (20.0)	2 (11.8)	9 (11.7)	0.50
Diabetes mellitus	2 (9.5)	1 (5.0)	3 (17.6)	13 (16.9)	0.43
Current smoking	4 (19.0)	8 (40.0)	4 (23.5)	34 (44.2)	0.10
Prior coronary artery disease	1 (4.8)	1 (5.0)	2 (11.8)	8 (10.4)	0.72
Prior stroke	0 (0.0)	1 (5.0)	1 (5.9)	9 (11.7)	0.16
Clinical and radiographic severity of SAH on admission					
WFNS grade	4 [3–5]	4 [2–5]	4 [2–5]	2 [1–2.5]	< 0.01
mFS grade	3 [3–3]	3.5 [3–4]	3 [3–4]	3 [3–3]	< 0.01
IVH	14 (66.7)	16 (80.0)	12 (70.6)	47 (61.0)	0.43
Hydrocephalus	17 (81.0)	16 (80.0)	12 (70.6)	45 (58.4)	0.11
Therapeutic intervention					
Coiling	8 (38.1)	6 (30.0)	7 (41.2)	22 (28.6)	0.69

Variables are presented as mean ± SD, median [interquartile range], or number (%)

*CE* cerebral edema, *IVH* intraventricular hemorrhage, *mFS* modified Fisher, *SAH* subarachnoid hemorrhage, *SEBES* subarachnoid hemorrhage early brain edema score, *WFNS* World Federation of Neurological Surgeons

^†^*p* value are calculated by Pearson chi-square test or Fisher’s exact test, or ANOVA test or Kruskal–Wallis test as appropriate

**Fig. 2:**
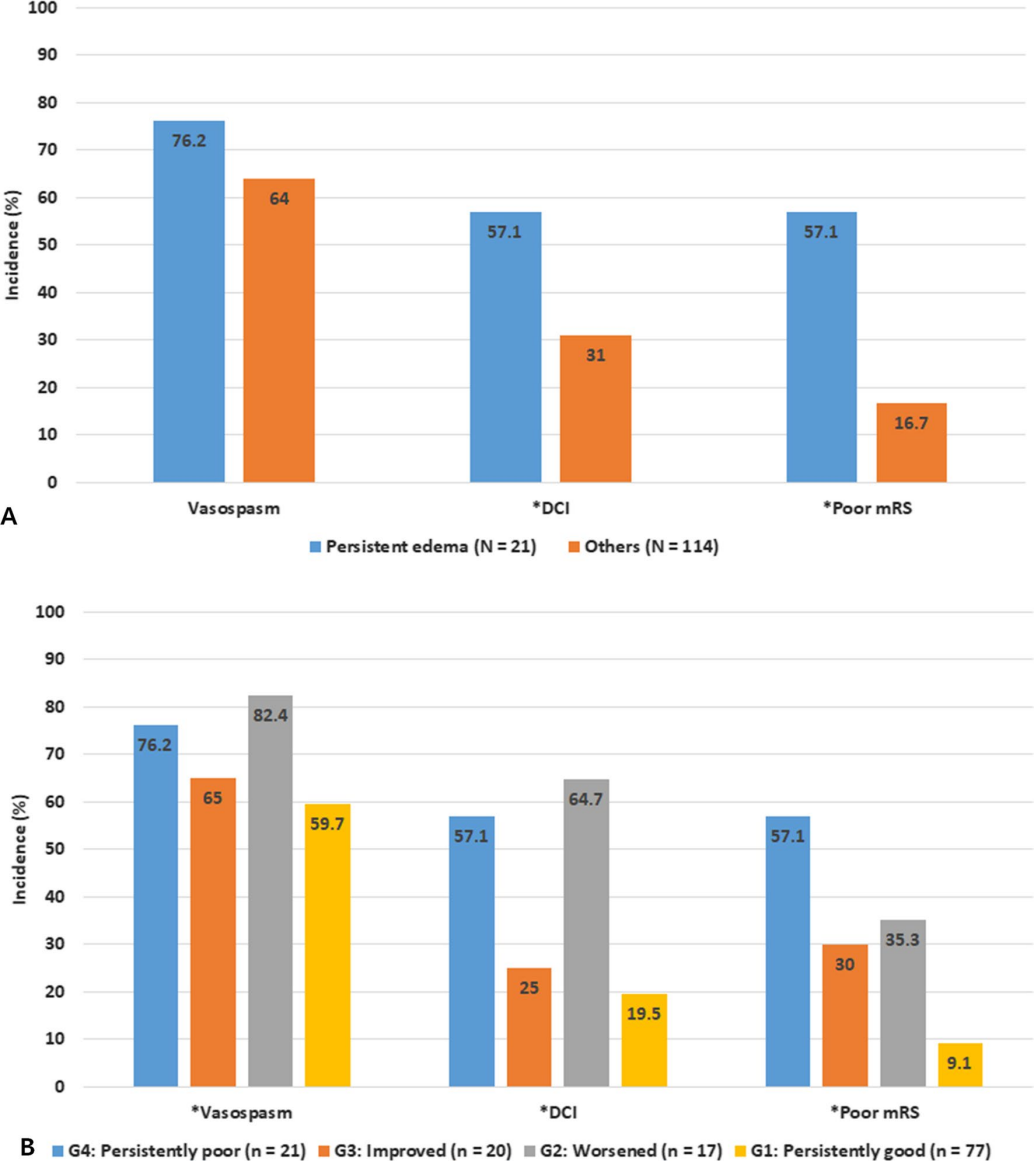
Clinical course according to the persistent CE (**A**) and change of CE (**B**). *CE* cerebral edema; *DCI* delayed cerebral ischemia, *mRS* modified Rankin scale. *P value < 0.05 was evaluated by Pearson chi-square test

Subjects with persistent CE showed a higher incidence of DCI and unfavorable functional outcome at 3 months after SAH compared to those without persistent CE. Furthermore, based on the change in CE, subjects with persistently poor or worsened SEBES grade had a higher incidence of DCI and unfavorable functional outcome compared to those with persistently good or improved SEBES grade (Fig. 2).

## Discussion

In this study, we investigated the acute peripheral immune response in SAH subjects according to the serial change in CE based on the SEBES grading. In patients who showed persistent CE, cytokines associated with eosinophilic activity including Eotaxin and inflammatory cytokine including IL-6 were persistently elevated from hyperacute to subacute stage of SAH, and regulatory cytokine such as sCD40L and inflammatory cytokine including TNF-α was transiently higher, especially at 6–8 days after stroke. Currently, the importance of pathophysiologic mechanisms in the very early phase after SAH, naming EBI, is being recognized as an important cause of adverse outcomes including DCI [25]. However, pathophysiologic mechanisms related to EBI including CE is largely unknown. Thus, serially measured cytokine data in humans can provide insight into peripheral immune response leading to brain tissue hypoxia, cerebral inflammation and blood–brain barrier (BBB) breakdown [3].

Interestingly, statistical significance of candidate cytokines contributing to persistent CE was dramatically changed by stepwise adjusting for covariables in multivariable logistic analysis as expected: at first, the OR of Eotaxin (at T1 and T4), sCD40L (at T4) and TNF-α (at T4) improved after adjusting basic epidemiologic information in model 1, compared to the results in univariable analysis. However, except for the Eotaxin (at T4), the OR of IL-6 (at T1 and T3), sCD40L (at T4) and TNF-α (at T4) diminished after additional adjusting radiographic and clinical severity of SAH in model 2, compared to the results in the first step multivariable analysis. This phenomenon demonstrates that strong correlation between degree of CE and basic epidemiologic information especially for age (results from model 1), and strong collinearity between specific cytokines such as inflammatory or immune-modulator cytokines and severity of SAH (results from model 2).

### Role of Eotaxin for persistent CE

In the present study, Eotaxin at T_1_ (within 24 h) and T_4_ (6–8 days) was identified as the strongest predictor for persistent CE, because Eotaxin was consistently elevated until subacute stage after SAH onset and its statistical significance was preserved in patients with persistent CE after controlling for all covariables even including radiographic and clinical grading for severity of SAH, especially at 6–8 days after SAH. Eotaxin-1 has been known to be primarily involved in recruitment of eosinophils and playing a role during allergy-related diseases as well as inflammatory disorders, such as atherosclerosis [26, 27]. Furthermore, Eotaxin can traverse the BBB and has been studied for its role in various range of neurodegenerative disease [28, 29]. In addition, primary cultures of astrocytes, pericytes, and microglia can be provoked to release CCL11 following exposure to inflammatory mediators in vitro [30, 31]. Thus, serum Eotaxin-1 level is a potential biomarker that may be valuable for evaluating the severity of CE beyond clinical and radiographic severity of SAH. It is interesting to think that activated eosinophilic immune response in peripheral blood across the BBB as effects centrally [32] in line with previous results showing a possible role of Eotaxin in poor functional outcomes after stroke [33, 34]. Furthermore, pertinent to our study, a recent study by Fenandez-Castaneda et al. examined systemic changes in COVID-19 patients and how systemic inflammation causes neurologic symptoms [35]. In fact, they found that systemic Eotaxin administration specifically caused hippocampal microglial reactive and impaired neurogenesis. This study among others shows the systemic elevations in systemic inflammation can have impacts on brain pathophysiology.

### Role of sCD40L and PDGF-AA for persistent CE

CD40 ligand (CD40L) and the soluble form of CD40L (sCD40L) are members of the TNF family and are expressed in a variety of cell types, including B cells, epithelial cells, fibroblasts, endothelial cells, and platelets, and exhibit proinflammatory and procoagulant effects [36, 37]. Previous studies also have found that serum CD40L levels are frequently elevated in patients with sepsis and are associated with mortality in these patients [38, 39]. Furthermore, recent studies have suggested that CD40L or sCD40L increases the permeability of the BBB [40]. CD40L works by binding to CD40, which is expressed on the surface of B cells and other cells, including macrophages, dendritic cells, smooth muscle cells, microglia, astrocytes, and endothelial cells which are also the components of the BBB. Our study also demonstrates that PDGF-AA tended to be associated with persistent CE even. Therefore, in line with previous results, platelet activation and the signaling pathways involved in hemostatic conditions with sCD40L [41] can be a possible pathomechanism of CE in SAH patients.

### Role of IL-6 and TNF-α for persistent CE

Our study demonstrates that IL-6 and TNF-α are also candidate cytokines contributing to persistent CE. Traditionally, pro-inflammatory cytokines, such as IL-6 and TNF-α as well as IL-1β and matrix metalloproteinases (MMPs), have been studied in a wide range of medical conditions including ischemic and hemorrhagic stroke as a surrogate for predicting poor outcomes [42–44]. Furthermore, in SAH patients, the IL-6 in the cerebral microdialysate as a marker for neuroinflammation has been shown to be associated with DCI and unfavorable outcome following aSAH [45, 46]. In our study, statistical significance of these inflammatory cytokine, especially IL-6, was dramatically diminished after additional adjustment of clinical and radiographic severity of SAH. This finding indicates a strong association between pro-inflammatory cytokine and severity of SAH which has been reported previously [24].

### Clinical course according to change of CE

In our study, patients with persistent CE had a higher clinical severity defined by WFNS grade, and developed a higher incidence of adverse events than those without persistent CE. Further grouping of subjects according to change in CE over time showed a more distinct clinical course. Subjects with improved SEBES showed a modest clinical course, compared to subjects with persistently poor or good SEBES grade. Subjects with progressive CE showed a worse clinical course, with outcomes equivalent to subjects with persistently poor SEBES grade (Fig. 2). In line with recent studies demonstrating the clinical importance of the evolving CE [12, 14], this phenomenon demonstrates the importance of CE.

## Limitation

This study is a single-center observational study and has a relatively small number of enrolled subjects. This can be a possible source of confounders and bias. Furthermore, subgroup analysis focusing on the dynamic pattern of serially measured cytokine according to change of CE may be more helpful to reveal the underlying mechanism of the CE, thus more data is needed to reveal a constant dynamic pattern of cytokine change at future. Second, only peripheral inflammation is examined. Examining central inflammation by analyzing cerebrospinal fluid and/or cerebral micro dialysis analysis could further our understanding of the underlying pathophysiological mechanisms. However, the explicit focus of our study is to study the systemic reaction to SAH. The hypothesis is that the peripheral reaction to SAH has an impact on central pathophysiologic processes. In that way, we have been studying the peripheral immune response in SAH patients and recognized their value for prediction of clinical course (i.e., functional outcome, mortality, DCI, etc.,) [23, 24, 47]. Thus, this study is a part of the extension of previous studies based on the evidence of biological plausibility of peripheral blood samples for investigating the systemic immune response in patients with SAH. These findings are in line with previous results focusing on the peripheral immune response in patients with central nervous system damage including SAH [48–50], TBI [51, 52], and ischemic stroke [44]. Thirdly, although we identify the main candidate contributing persistent CE, such as Eotaxin, future study is needed focusing on the overall immune reaction based on cytokine interaction to improve practical application of immune modulating treatment in SAH patients. Until now, there are preliminary studies to investigate the possible utility of immune modulating agents including epoxyeicosatrienoates (endogenous regulators of neuroinflammation and cerebral blood flow) [53], dapsone (considering neuroprotective effect via anti-inflammatory mechanism) [54], stellate ganglion block (inhibiting the inflammatory response during EBI and by reducing endothelial dysfunction and relieving vasospasm) [55], cerebrolysin (brain-specific proposed pleiotropic neuroprotective agent) [56], subcutaneous IL-1Ra (anti-inflammatory reaction focusing on the inflammation mediated by the cytokine IL-1 as a IL-1 receptor antagonist) [57, 58] and SB203580 (anti-inflammatory reaction by the inhibition of TNF-a) [59], beyond traditional anti-inflammatory agents (i.e., steroid [60], or non-steroidal ant-inflammatory drug [61]). However, most of the clinical trials focusing on the utility of immune-modulating agent in SAH patients failed to be adopted in clinical field. These results indicate the limitation of immune-modulating agent focusing on the specific cytokine after SAH due to the complexity of immune reaction in these patients. Previously, we also found that abnormally elevated proinflammatory cytokine (i.e., IL-6, TNF-a) was combined with elevation of anti-inflammatory cytokine (i.e., IL-8 and IL-1R) in patients with poor outcome after SAH based on the detection of a unique cytokine cluster [24]. Thus, in the future study, the cluster and network analysis will be helpful for revealing the complex mechanism of development of the persistent CE which may improve the development of the immune modulating strategy to prevent devastating CE in SAH patients.

## Conclusions

We identified serum cytokines at different time points that were independently associated with persistent CE after SAH. The disruption in immune activity at the acute phase of SAH persisted through the subacute phase as evidenced by the complex cytokine interactions captured through network analysis. This study is an important step towards an integrated approach to describe global inflammatory reaction after SAH.

## Supplementary Information

**Additional file 1.** Cytokine analysis.

## Data Availability

All data used and analyzed for the current study are available from the corresponding author on reasonable request.

## Abbreviations

BBB: Blood–brain barrier
CE: Cerebral edema
CI: Confidence interval
CT: Computed tomography
DCI: Delayed cerebral ischemia
EBI: Early brain injury
IVH: Intraventricular hemorrhage
mFS: Modified Fisher score
MRI: Magnetic resonance imaging
mRS: Modified Rankin Scale
OR: Odds ratio
SAH: Subarachnoid hemorrhage
SEBES: Subarachnoid hemorrhage early brain edema score
WFNS: World Federation of Neurosurgical Societies
