# Supplementary material for: Systemic inflammatory markers of persistent cerebral edema after aneurysmal subarachnoid hemorrhage
Source: J Neuroinflammation. 2022 Aug 4;19:199. doi: 10.1186/s12974-022-02564-1 (PMC9354324; doi:10.1186/s12974-022-02564-1)
Supplement: Supplementary file 1 — Additional file 1. Cytokine analysis. [file 12974_2022_2564_MOESM1_ESM.docx]

**Cytokine analysis**

Blood samples were collected in K2 EDTA vacutainer tubes and centrifuged within an hour of draw (1460 x g for 10 minutes at 4°C) generating plasma. Plasma was centrifuged a second time (1460 x g for 10 minutes at 4°C) in order to generate platelet poor plasma. Platelet poor supernatant was collected and stored at -80°C until ready for use. Cytokine sample concentrations were determined using a MAGPIX magnetic bead based ELISA 17-plex assay (Millipore) to test for the following cytokines: the CC chemokines (MCP1/CCL2, Rantes/CCL5, Eotaxin/CCL11), the macrophage inflammatory proteins (MIP-1α and MIP-1β), the interleukin-1 superfamily (IL1-Ra, IL-6), interleukin-8 (IL-8), interleukin-10 (IL-10), the platelet derived factors (PDGF-AA and PDGF-AB/BB), tumor necrosis factors (TNF-α), the soluble CD40-ligand (sCD40L), colony stimulating factors (GM-CSF/CSF2 and GCSF/CSF3), interferon γ-induced protein 10 (IP-10), type II interferon family (IFN-γ). Plasma was analyzed using a multiplex assay, which utilizes fluorescent dye-coated magnetic beads (MAGPIX magnetic bead based ELISA 17-plex assay [Millipore]) and a magnetic bead plate reader (Luminex MagPix [Luminex, Austin, TX]), according to manufacturer's instructions. The plate reader reports results as fluorescence intensity. Mean fluorescence intensity was calculated from the area on the Luminex assay. Per manufacturer's instructions, plasma was diluted 1:100 for measurement of CCL5, PDGF-AA, PDGF-AB/BB and undiluted plasma was used for the 17plex (25ul/well). Unknowns were compared to serial dilutions of manufacturer provided standards and controls. Assays were run in duplicate. In short, samples were incubated in 96 well plates overnight with premixed beads on an agitator at 4°C) and plates were washed. Detection antibodies were added to each well for a one hour incubation period followed by the addition of streptavidin-phycoerythrin and another 30 minutes of incubation. Plates were washed and the beads were re-suspended in drive fluid and the plate was run on the MAGPIX with xPONENT software. All cytokine values were expressed in pictograms per milliliter (pg/ml).
